# Immature Dengue Virus: A Veiled Pathogen?

**DOI:** 10.1371/journal.ppat.1000718

**Published:** 2010-01-08

**Authors:** Izabela A. Rodenhuis-Zybert, Hilde M. van der Schaar, Júlia M. da Silva Voorham, Heidi van der Ende-Metselaar, Huan-Yao Lei, Jan Wilschut, Jolanda M. Smit

**Affiliations:** 1 Department of Medical Microbiology, Molecular Virology Section, University Medical Center Groningen and University of Groningen, Groningen, The Netherlands; 2 Department of Microbiology and Immunology, College of Medicine, National Cheng Kung University, Tainan, Taiwan; University of Washington, United States of America

## Abstract

Cells infected with dengue virus release a high proportion of immature prM-containing virions. In accordance, substantial levels of prM antibodies are found in sera of infected humans. Furthermore, it has been recently described that the rates of prM antibody responses are significantly higher in patients with secondary infection compared to those with primary infection. This suggests that immature dengue virus may play a role in disease pathogenesis. Interestingly, however, numerous functional studies have revealed that immature particles lack the ability to infect cells. In this report, we show that fully immature dengue particles become highly infectious upon interaction with prM antibodies. We demonstrate that prM antibodies facilitate efficient binding and cell entry of immature particles into Fc-receptor-expressing cells. In addition, enzymatic activity of furin is critical to render the internalized immature virus infectious. Together, these data suggest that during a secondary infection or primary infection of infants born to dengue-immune mothers, immature particles have the potential to be highly infectious and hence may contribute to the development of severe disease.

## Author Summary

Dengue virus represents a major emerging arboviral pathogen circulating in the (sub)tropical regions of the world, putting 2.5 billion people at risk of infection. Each of the four circulating serotypes can cause disease ranging from febrile illness to devastating manifestations including dengue hemorrhagic fever and dengue shock syndrome. Severe illness is observed in individuals experiencing a re-infection with a heterologous dengue virus serotype and in infants born to dengue-immune mothers, presumably due to antibody-dependent enhancement of infection. Interestingly, it has been recently reported that patients experiencing a secondary infection have elevated levels of antibodies directed against the prM protein of immature dengue virus particles. Although it is known that cells infected with dengue virus release substantial amounts of prM-containing virions, numerous functional studies have demonstrated that immature particles lack the ability to infect cells. Herein, we show that essentially non-infectious fully immature dengue virions become virtually as infectious as wild type virus particles in the presence of prM antibodies. Anti-prM antibodies facilitate efficient binding and entry of immature dengue virus into cells carrying Fc-receptors. Furthermore, furin activity in target cells is critical for triggering infectivity of immature virus. These data indicate that immature dengue virus has the potential to be highly infectious and hence may contribute to disease pathogenesis.

## Introduction

Dengue virus (DENV) represents a major emerging arthropod-borne pathogen. There are four distinct serotypes of DENV which, according to WHO estimates, infect about 50-100 million individuals annually, mostly in the (sub)tropical regions of the world. While most DENV infections are asymptomatic or result in self-limited dengue fever (DF), an increasing number of patients present more severe, potentially fatal clinical manifestations, such as dengue hemorrhagic fever (DHF) and dengue shock syndrome (DSS). It is well established that a major risk factor for the development of DHF/DSS is secondary infection with a heterotypic virus serotype [1]–[3]. Also primary infection of infants born to dengue-immune mothers may lead to severe disease [1],[4],[5]. These observations have led to the hypothesis of antibody-dependent enhancement (ADE) of infection [3],[6],[7]. Increased disease severity appears to correlate with high circulating virus titers [3], [8]–[11], suggesting that antibodies directly influence the infectious properties of the virus. The molecular mechanisms by which antibodies enhance DENV infection however remain elusive.

DENV, as well as other major human pathogens like West Nile virus (WNV), yellow fever virus, and tick-borne encephalitis (TBEV) belong to the Flavivirus genus within the family *Flaviviridae*. Flaviviruses enter cells via clathrin-mediated endocytosis and fuse from within acidic endosomes, through which the viral genome gains access to the target cell cytoplasm[12]. Following RNA replication and protein translation, immature virions, which contain heterodimers of the transmembrane proteins E and a precursor form of M (prM), are assembled within the ER. Subsequently, the particles mature by passing through the Golgi and trans-Golgi network (TGN) [13]. In the acidic environment of the TGN, the virion undergoes a conformational change and the cellular endoprotease furin cleaves prM into M and a peptide (“pr”) that remains associated with the virion [14]. Upon release, the pr peptide dissociates from the virion, resulting in the formation of mature progeny virions.

Cells infected with DENV secrete high levels (∼30%) of prM-containing immature particles [15],[16] suggesting that cleavage of prM to M is not efficient. These DENV particles are released from infected cells as fully immature prM-containing particles and partially immature particles containing both prM and M proteins in the viral membrane [17]. Extensive functional analyses have revealed that fully immature flaviviruses lack the ability to infect cells, as the presence of uncleaved prM in the virion blocks the E glycoprotein from undergoing the pH-induced conformational changes that are required for membrane fusion [16], [18]–[22]. Although immature particles are therefore generally considered as irrelevant by-products of infected cells, the rates of prM antibody responses are significantly higher in patients with secondary infection compared to those with primary infection [23]. Furthermore, previous reports show that prM antibodies can enhance DENV infection. Enhancement of infection was observed for wild-type virus [24],[25], presumably due to the presence of uncleaved prM in these preparations, and with DENV particles containing high levels of prM generated from cells treated with chloroquine [26]. It is thus quite puzzling if indeed the presence of prM obstructs DENV infectivity, how immature particles contribute to disease pathogenesis and what role do anti-prM antibodies play in the enhancement of infection? The present study addresses these questions.

## Results

### Fully immature dengue virus particles becomes highly infectious in the presence of prM antibodies

First, we investigated if prM antibodies are able to render fully immature DENV infectious. To this end, immature DENV-2 strain 16681 particles were produced in furin-deficient LoVo cells. We have used this procedure before and showed that LoVo-derived particles have an average content of 94%±9% prM [16]. Furthermore, we demonstrated that the specific infectivity of LoVo-derived fully immature DENV is at least 10,000-fold reduced compared to that of wild-type virus on cells highly permissive to infection [16]. The infectious properties of fully immature DENV virions were determined in Fc-receptor-expressing K562 cells in the absence or presence of increasing concentrations of the 70–21 antibody. This is an IgG2a antibody that has been isolated from DENV-infected mice and is mapped to amino acids 53-67 of prM [25]. Antibodies recognizing this epitope are abundantly present in sera of DHF/DSS patients [16],[27]. K562 cells were infected with DENV at a multiplicity of 100 genome-containing particles per cell (MOG 100). The number of genome-containing particles (GCP) was determined by quantitative PCR analysis of reverse-transcribed viral RNA [15]. At 24–48 hours post-infection (hpi), cells were fixed and prepared for flow-cytometric analysis to determine the number of infected cells, measured on the basis of dengue E protein expression. We observed that 43 hpi is optimal for read-out as it represents a single round of infection together with a high mean fluorescence intensity per infected cell (Fig. S1).

**Figure 1:**
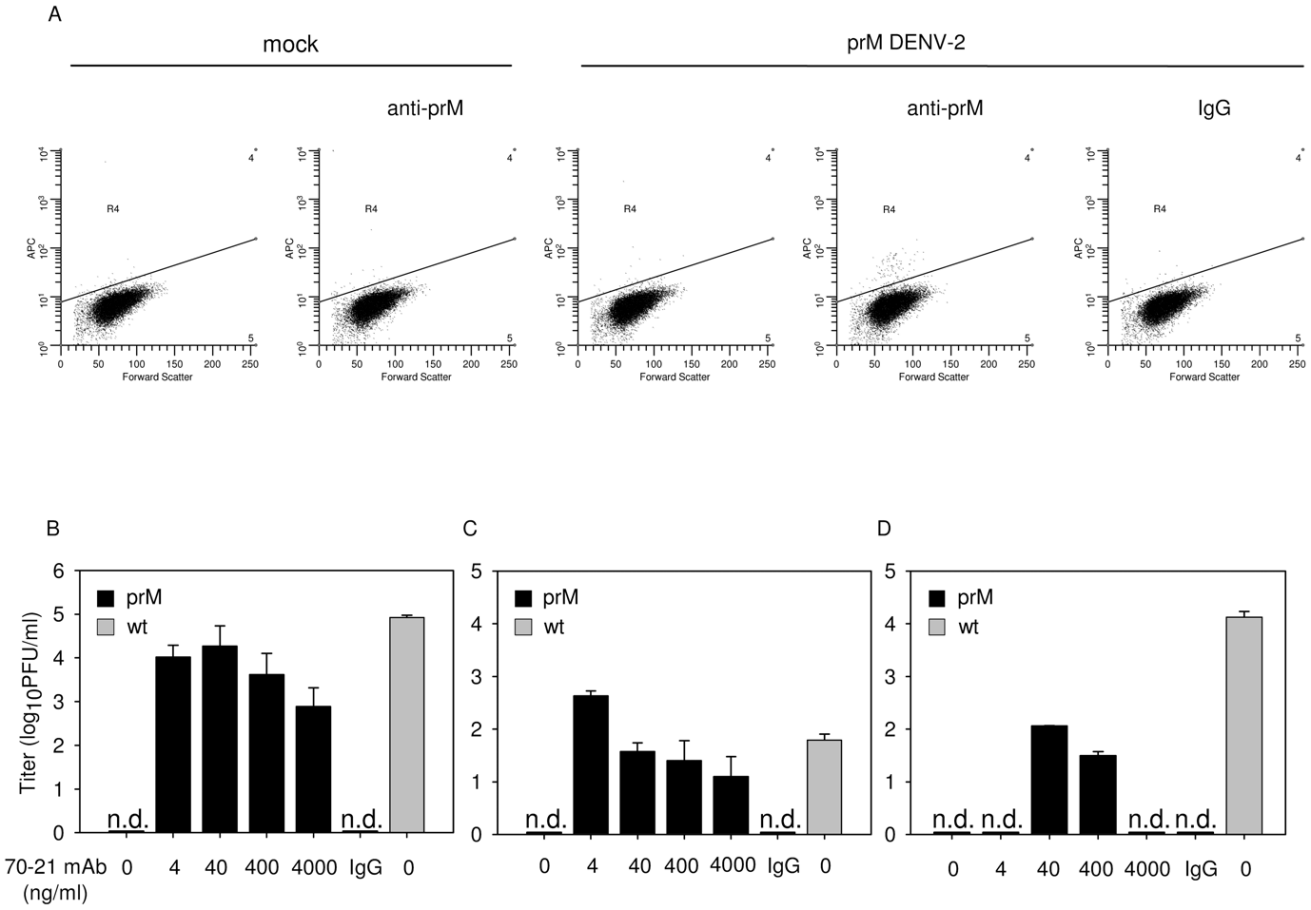
Immature DENV particles become highly infectious in the presence of anti-prM antibodies. K562 cells were infected with immature (prM) or wild-type (wt) DENV-2 strain 16681 at MOG 100 in the presence or absence of anti-prM 70–21. (**A**) Representative read-out of the percentage of infected cells. Prior to infection, immature DENV particles were incubated with 40 ng/ml 70–21 antibody for 1 h at room temperature. At 43 hpi, the cells were fixed, stained intracellularly with Alexa-647-coupled anti-E antibody 3H5.1, and subjected to flow-cytometric analysis. Mock-infected cells and an IgG isotype control (murine IgG2a) antibody were used as controls. Titrations of the virus particles released at 43 hpi from infected K562 cells (**B**), U937 cells (**C**), and primary human PBMCs (**D**) were performed by plaque assay on BHK-15 cells. Data are expressed as means of at least three independent experiments. The error bars represent standard deviations (SD); (n.d.) denotes “not detectable”.

In agreement with our previous study [15],[16], we observed that fully immature DENV particles are essentially non-infectious as the number of E-positive cells did not exceed the limit of detection (Fig. 1A). Remarkably however, substantial numbers of E-positive cells were observed upon infection of cells with fully immature particles opsonized with the anti-prM antibody (Fig. 1A). Subsequent titration of the cell supernatants at 43 hpi revealed that opsonization of immature DENV with anti-prM antibody dramatically enhanced (up to 30,000-fold) virus particle production (Fig. 1B). The results show that prM antibodies render essentially non-infectious immature DENV nearly as infectious as wild-type virus (Fig. 1B). Enhancement of immature DENV infectivity was seen in a broad antibody concentration range, even at conditions of high antibody excess.

To ensure that the observed high level of enhancement is not restricted to a single antibody we performed additional experiments with the murine IgG2a prM antibody 2H2 [28]. The results show that 2H2 stimulated the infectious properties of fully immature particles up to 1,000 fold (Fig. S2A). Although, this antibody has previously been shown to enhance DENV infectivity [26], the power of enhancement observed here is striking and demonstrates that prM antibodies render essentially non-infectious fully immature DENV highly infectious.

Subsequently, we investigated the enhancing properties of both prM antibodies in Fc-receptor-bearing human monocytic U937 cells and observed that the antibodies again significantly stimulate the infectivity of immature particles (Fig. 1C, S2B). Thereafter, we studied the infectious properties of immature DENV particles in primary human PBMCs, cells which are known to be involved in dengue pathogenesis. The results show that, also under these conditions, prM antibodies render fully immature particles infectious (Fig. 1D, S2C).

### Antibodies against prM facilitate binding of immature dengue virus particles to FcγII-receptors on target cells

**Figure 2:**
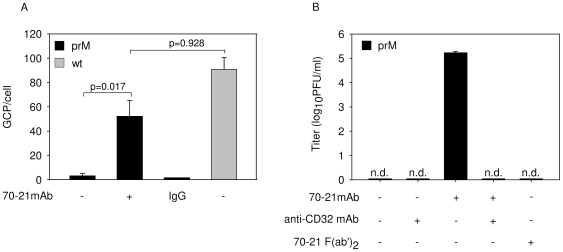
Anti-prM antibody stimulates binding of immature DENV particles to cells through interaction with FcγIIR. (**A**) Binding of immature and wild-type virions with and without prior opsonization to antibodies (40 ng/ml) to K562 cells. Virus-cell binding was measured after 1 h incubation at 4°C by q-PCR analysis. (**B**) Effect of anti-CD32 mAb and 70–21 F(ab')_2_ fragments on virus particle production. Virus particle production was determined as described in the legend to Figure 1B. Data are expressed as means and SD of three independent experiments. Two-tailed Student's t-tests were performed for statistical analysis of the data.

To better understand the mechanism by which prM antibodies trigger infectivity of immature DENV, we analyzed the distinct steps in the cell entry pathway of the virus. First, the binding of immature virions to K562 cells was determined by quantitative-PCR. In order to determine the number of bound GCP per cell, the amount of virus added per cell was increased 10-fold compared to the concentration used in the infectivity experiments. The results show that antibody-opsonized immature DENV binds approximately 30-fold more efficiently to cells than immature particles in the absence of antibody (Fig. 2A). Indeed, immature particles opsonized with anti-prM bound almost as efficiently to cells as wild-type DENV in the absence of antibody. Moreover, immature DENV particles failed to interact efficiently with baby hamster kidney cells (BHK-15), cells which are highly permissive for dengue infection (data not shown) suggesting that the observed lack of infectivity is partially related to the poor binding efficiency of immature particles to cells. It is likely that binding of virus-antibody complexes is mediated by direct interaction of the antibody with the Fc-receptor expressed on the cell surface. Indeed, treatment of cells with an anti-CD32 antibody to block FcγII-receptor interaction, or opsonization of particles with mAb70-21 F(ab')_2_ fragments severely reduced virus particle production upon infection of K562 cells with opsonized immature virions, whereas it had no effect on infection with wild-type virus (Fig. 2B). Although this antibody has been previously described to enhance the infectious properties of wild-type DENV in cells with or without Fc-receptors [25],[27], clearly in the case of immature particles interaction with the Fc-receptor is important for infectivity. Taken together, these data indicate that prM antibodies facilitate efficient interaction and cell entry of virus-immune complexes via the FcγII-receptor.

### Furin activity is critical in rendering immature particles infectious

**Figure 3:**
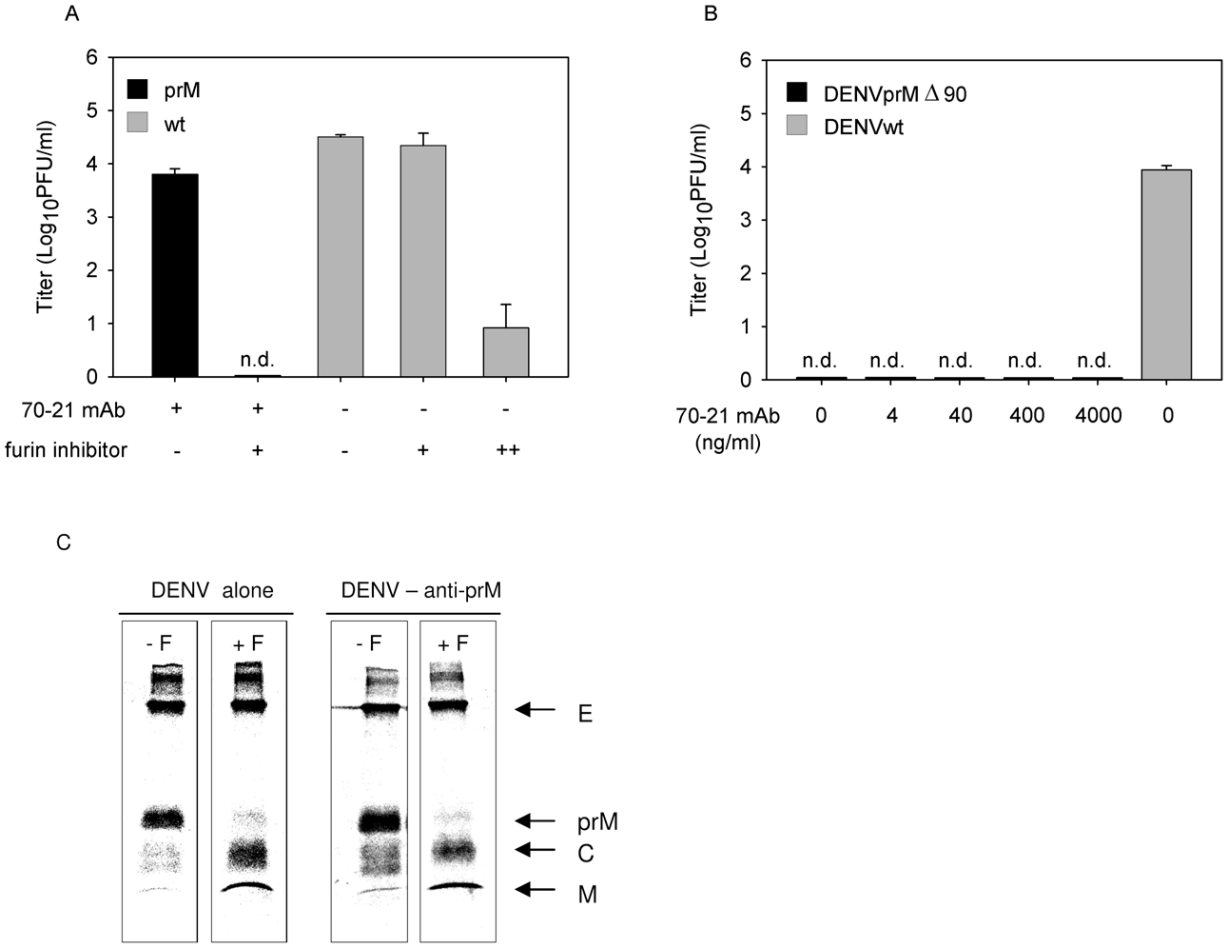
Immature DENV particles mature upon FcγIIR-mediated cell entry. (**A**) K562 cells were infected with DENV or DENV-immune complexes in the presence or absence of furin inhibitor (25 µM). As a positive control for compound activity, wild-type DENV-infected cells were treated with an additional dose of furin inhibitor at 24 hpi to impede virus maturation and consequently the production of infectious virions (++). Virus particle production was determined as described in the legend to Figure 1B. Data are expressed as means and SD for three independent experiments; (n.d.) denotes “not detectable”. (**B**) K562 cells were infected with DENVprMΔ90 at MOG 100 in the presence of increasing concentrations of prM antibody 70–21. As control, the infectivity of wild-type virus (generated from the infectious clone pD2/IC-30P) at MOG 100 is shown. Virus production was assessed as described in the legend to Figure 1B. (**C**) prM antibodies do not affect cleavage maturation of DENV particles. Purified [^35^S]methionine-labeled immature particles with and without enhancing concentrations of anti prM mAb 70–21 were incubated with furin at pH 6 for 16 h. Next, viral protein composition was analyzed by non-reducing SDS-polyacrylamide gel electrophoresis.

Efficient FcγIIR-mediated cell entry does not however clarify what is the trigger for immature virions to become infectious, since the presence of prM has been shown to obstruct membrane fusion activity of the virus [14],[16],[18]. One could speculate that anti-prM antibody bound to immature virions induces a conformational change that would enable the E protein to trigger membrane fusion irrespective of the presence of prM. Another scenario might be that prM-containing virions mature upon cell entry since furin, although predominantly present in the TGN, also shuttles between early endosomes and the cell surface. To verify the potential involvement of furin during virus cell entry, we investigated the infectious properties of antibody-opsonized immature DENV in cells treated with furin inhibitor, decanoyl-L-arginyl-L-valyl-L-lysyl-L-arginyl-chloromethylketone (decRRVKR-CMK). In aqueous solution, decRRVKR-CMK has a half-life of 4–8 h [29] and therefore it is not expected to interfere with the maturation process of newly assembled virions within the infected cell. The results show that inhibition of furin activity completely abrogated virus particle production in cells infected with antibody-opsonized immature virions, whereas infection of cells with wild-type virus remained unaffected under these conditions (Fig. 3A).

To further substantiate the role of furin in triggering viral infectivity, we generated a furin cleavage-deficient virus (pDENprMΔ90) by deletion of the lysine on the position 90 (87-R-R-E-K-R-91) within the furin recognition sequence. Subsequently, DENVprMΔ90 virus and wild-type DENV-2 16681 (generated from pD2/IC-30P) virus were produced by transfection of RNA transcripts derived from the cDNA plasmids into BHK-15 cells. Virus production was measured by determining the number of physical particles based on GCP and the number of infectious units as measured by plaque assay. The presence of physical particles was further evaluated in three-layer ELISA experiments, by coating plates with a similar number of genome-containing DENVprMΔ90 particles and LoVo-derived immature particles. Similar OD values were measured for DENVprMΔ90 and LoVo-derived viruses (data not shown), which confirms the presence of physical particles and suggests that the number of genome-containing particles is accurately determined. Subsequent titration studies revealed that the specific infectivity of DENVprMΔ90 mutant virus is reduced by a factor of 12.000 compared to that of wild-type virus (generated from pD2/IC-30P) and is comparable to LoVo-derived immature virus. Next, K562 cells were infected with DENVprMΔ90 mutant virus opsonized with increasing concentrations of prM antibody 70–21. Figure 3B shows that disruption of the furin-recognition motif within the prM protein of the virus abrogates the enhancing activity of the anti-prM-antibody, demonstrating that enzymatic cleavage of prM to M by furin is critical to render immature DENV infectious.

To address the question as to whether prM to M cleavage can occur upon interaction of immature DENV particles with antibodies, we incubated ^35^S-methionine-labeled immature particles in the absence and presence of antibodies with exogenous furin for 16 h at pH 6.0 [16]. Protein visualization was done by SDS-PAGE analysis. In agreement with previous studies, we observed that exogenous furin treatment induces efficient cleavage of prM to M (Fig. 3C). Importantly, we found that the presence of prM antibodies does not affect DENV maturation, as virtually complete cleavage of prM to M was observed (Fig. 3C).

### Antibody-mediated entry of immature dengue virus particles does not lead to an increased production of virus particles per cell

**Figure 4:**
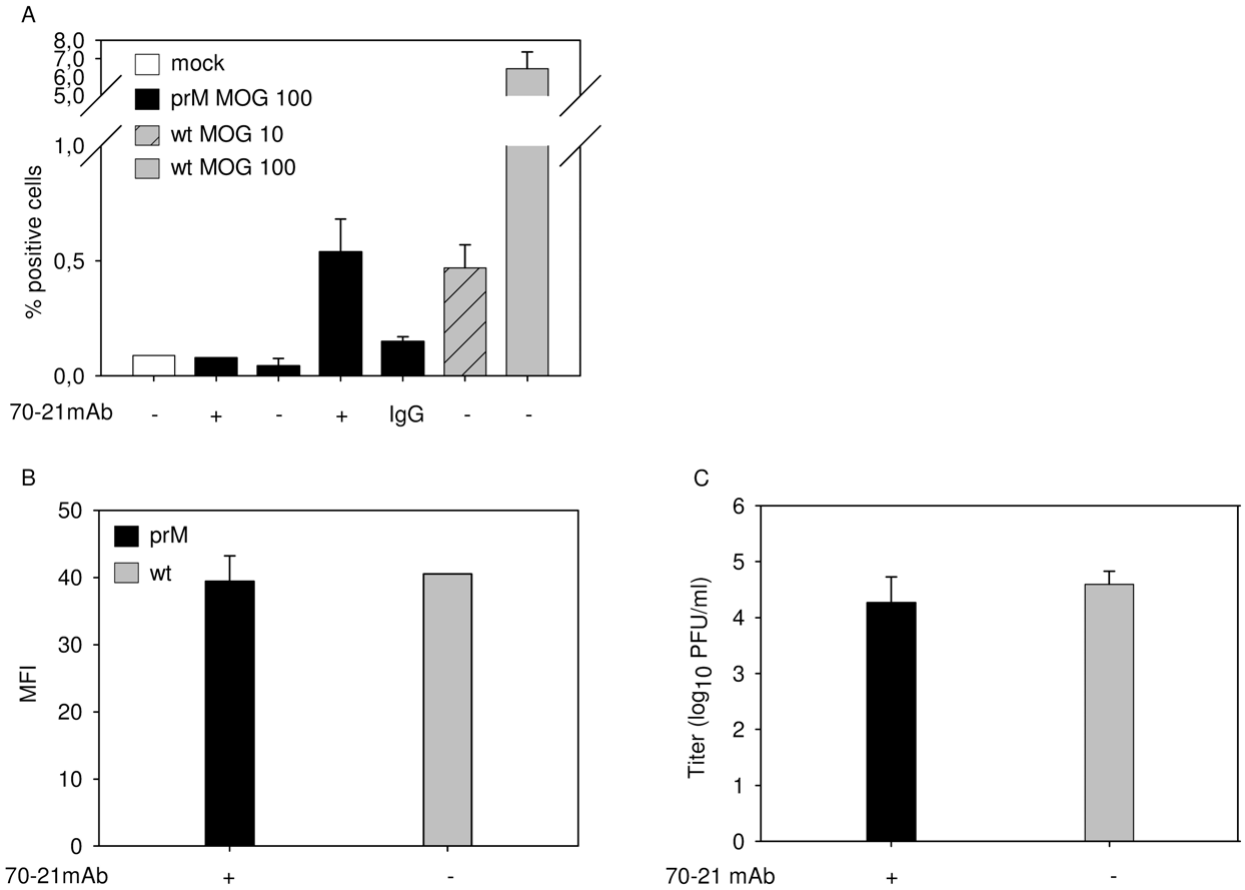
FcR-mediated entry of immature DENV does not enhance the number of progeny virions produced per cell. K562 cells were infected with immature (prM) DENV-2 at MOG 100 in the absence or presence of 40 ng/ml of 70–21 antibody and with wild-type virus at MOG 10 and 100. (**A**) At 43 hpi, the cells were fixed, stained intracellularly with Alexa-647-coupled anti-E antibody 3H5.1, and subjected to flow-cytometric analysis. Mock-infected cells and an irrelevant murine IgG2a antibody were used as controls. (**B**) Mean fluorescent intensity of cells infected with immature virus (MOG 100) and wild-type DENV (MOG 10) (**C**) Titration of the virus particles released at 43 hpi from infected K562 cells using plaque assay. Data are expressed as means of at least three independent experiments. The error bars represent standard deviations (SD).

It has been postulated that antibody-mediated entry of DENV leads to a higher production of virus particles per infected cell, a phenomenon often referred to as intrinsic ADE [30]. In this part of the study, we investigated whether prM-mediated entry of immature DENV supports intrinsic ADE. Since immature particles are essentially non-infectious in the absence of antibodies, we compared the production of prM-opsonized immature DENV particles with wild-type virus in K562 cells. For accurate comparison, we first searched for a condition that gives a similar percentage of infected cells. Infection of K562 cells with prM-opsonized immature DENV at a MOG of 100 leads to 0.53%+/−0.14 infected cells (Fig. 1, 4A). Comparable numbers of infected cells were detected for wild-type DENV at MOG 10 (Fig. 4A). Under these experimental conditions, no differences were observed in E protein expression and production of virus particles (Fig. 4B–C), which indicates that the presence of prM antibodies, while evidently stimulating the infectious properties of immature virions, has no enhancing effect on the number of progeny virions produced per cell.

### Antibody against prM enhances the infectivity of wild-type dengue virus in a furin-dependent manner

**Figure 5:**
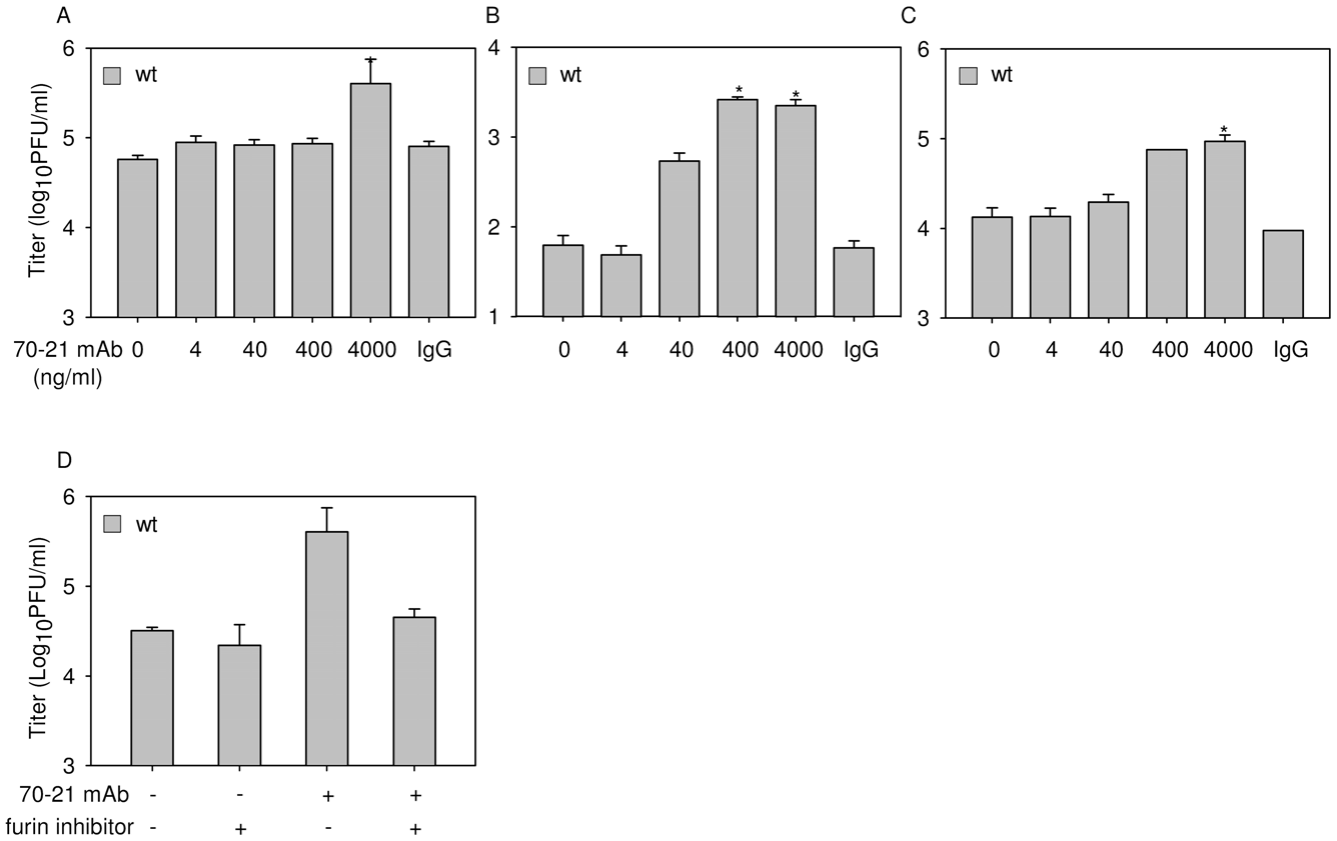
prM-specific antibody 70–21 enhances infectivity of wild-type DENV in a furin-dependent manner. Cells were infected with wild-type (wt) DENV-2 at MOG 100 in the presence of increasing concentrations of anti-prM 70–21. Virus particle production was measured at 43 hpi by plaque assay on BHK-15 cells. (**A**) K562 cells, (**B**) U937 cells, (**C**) PBMCs. (**D**) Enhancement of infection is dependent on endogenous furin activity. K562 cells were infected with DENV with and without prior opsonization with 4000 ng/ml 70–21 in the presence or absence of furin inhibitor as described in the legend to Figure 3. Data are expressed as means of at least three independent experiments. The error bars represent standard deviations (SD); (n.d.) denotes “not detectable”; * denotes significance (p<0.05) analyzed using Two-tailed Student's t-tests.

Given the high number of prM-containing particles in wild-type DENV preparations it is possible that prM antibodies also enhance the infectious properties of wild-type DENV. Indeed, in agreement with previous studies, opsonization of wild-type virus with prM antibodies results in a significant increase of viral infectivity (Fig. 5A-C, Fig. S3) [24],[25]. The level of enhancement is dependent on the cell type used and comparable to what has been described before for E antibodies [31],[32]. Enhancement of wild-type DENV infection was observed at higher antibody concentrations compared to that of immature particles. Although we do not completely understand these differences, we think that this may be related to the presence of structurally distinct immature virus particles (individual variations in prM/M content) in wild-type preparations [17]. Importantly, no enhancement of infection was observed in cells treated with furin inhibitor (Fig. 5D), demonstrating that furin activity in the target cells plays a vital role in triggering the infectious properties of antibody-opsonized immature particles in wild-type DENV preparations. Collectively, these results illustrate that prM antibodies enhance the infectious properties of prM-containing particles in wild-type DENV preparations and therefore may be important in disease pathogenesis.

### Dengue-immune sera enhances the infectious properties of immature dengue virus particles

**Figure 6:**
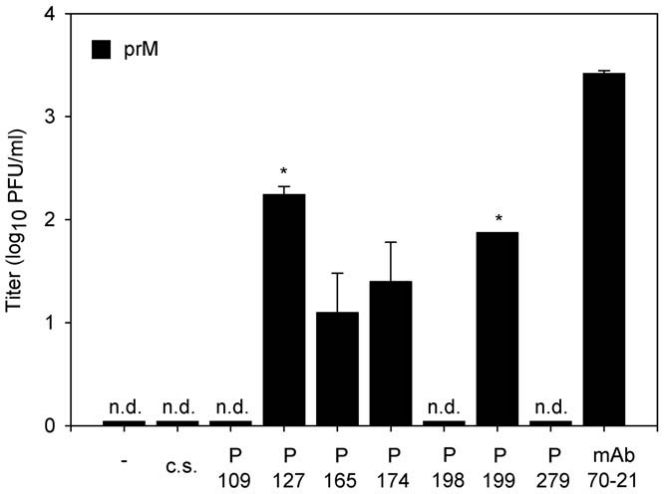
Immature DENV becomes infectious in the presence of DENV-immune sera. U937 cells were infected with immature (prM) DENV-2 at MOG 100 in the presence of 10-fold sequential dilutions of dengue patients sera. Virus particle production was measured at 43 hpi by plaque assay on BHK-15 cells. Sera of two patients showed significant enhancing activity towards immature DENV virions at a 10^4^ sera dilution. The error bars represent standard deviations (SD); (c.s.) denotes “control serum”; (n.d.) denotes “not detectable”; * denotes significance (p<0.05) analyzed using Two-tailed Student's t-tests.

As a first step towards elucidation of the implications of our findings in disease pathogenesis we evaluated the enhancing properties of 7 convalescent serum samples from patients infected with DENV-2. The infectious properties of immature particles opsonized with various dilutions of polyclonal sera were determined in U937 cells, since this cell line expresses both Fc receptors CD32 and CD64 on the cell surface. At 43 hr post-infection, the medium was harvested and the production of virus particles was measured by plaque assay. No plaques were found in the absence of sera and in the presence of DENV-naïve serum (Fig. 6). Convalescent sera from two distinct DENV-2 infected patients significantly enhanced the infectious properties of immature DENV particles at a 10,000 dilution (Fig. 6). Sera from two other patients enhanced the infectivity of immature particles to a minor extent as only a low number of plaques (average of 1.5 plaques) was observed. The three remaining patient sera did not show any effect on viral infectivity of immature particles as no viral plaques were observed. As expected, nearly all of the analyzed patient sera enhanced the infectious properties of wild-type virus particles (Fig. S4).

## Discussion

Multiple studies have shown that immature particles are non-infectious, the presence of prM obstructing the low-pH-induced conformational changes in the viral E glycoprotein required for membrane fusion of the virus [14],[16],[18],[21],[22],[33]. On the other hand, prM antibodies have been shown to enhance DENV infection [24],[25]. In this report, we show that the lack of infectivity of fully immature particles in the absence of antibodies is primarily related to inefficient binding of immature virions to the cell surface. If binding is facilitated through anti-prM antibodies, immature DENV particles become highly infectious presumably due to efficient intracellular processing of prM to M by the endoprotease furin.

Maturation upon entry has been previously reported for other enveloped viruses. Zhang and co-workers [34] showed that the infectivity of immature particles of Semliki Forest virus, an alphavirus, can be triggered by furin during viral endocytosis. It is likely that DENV maturation also occurs within acidic endosomes, since previous *in vitro* experiments have revealed that cleavage of immature particles by furin is dependent on the exposure of the virus to low pH [16]. We propose that the acidic conditions of the endosome, similar to those in the acidic TGN during processing of newly assembled virions, triggers an initial conformational change in the virion such that furin is able to cleave prM to M and the “pr” peptide. Interestingly, a recent report has shown that upon cleavage of prM a large fraction of pr peptide remains associated with the virion and that back-neutralization to pH 8.0 is required to release the pr peptide from the virion [14]. The authors interpreted this as a mechanism preventing newly assembled cleaved virions from undergoing membrane fusion in the acidic TGN. However, this notion is difficult to reconcile with our present observations, since virions that have matured within acidic endosomes of target cells do not return to neutral-pH conditions before initiating infection. One may speculate that the pr peptide stabilizes the E protein to such an extent that it survives the mildly acidic lumen of the TGN (∼pH 6.0), but is released at the more acidic pH of endosomes (∼pH 5.0) such that the E proteins have the capacity to rearrange to their fusion-active conformation. Another possibility is that upon cleavage of prM the pr peptide associates with the prM antibody instead of the E protein, thereby enabling the E proteins to adopt the fusion-active conformation.

The observed infectious potential of immature DENV virions in the presence of anti-prM antibodies may have important implications for our understanding of the processes involved in dengue pathogenesis. We speculate that in the early stages of a primary infection, before the appearance of virus-specific antibodies, immature virions would fail to penetrate host cells and therefore are of minor significance in disease development. On the other hand, during a secondary infection or primary infection of infants born to dengue-immune mothers, immature particles may become highly infectious due to the presence of anti-prM antibodies and hence may contribute to an increased dengue-infected cell mass and a high circulating virus titer, one of the preludes for the development of severe disease symptoms [3], [8]–[11]. Importantly, anti-prM antibodies may activate the infectious properties of a large population of virus particles, since we recently observed that a typical DENV-2 preparation of the prototype strain 16681 contains as much as 30% prM [16]. Taken together, our results suggest that immature DENV particles act as a veiled pathogen and can, like mature DENV contribute to the disease pathogenesis.

Variable levels of enhancement were seen with DENV-immune sera. As expected, virtually all of analyzed DENV-immune sera stimulated the infectivity of wild-type DENV. Interestingly, sera from 2 out of 7 patients significantly enhanced the infectious properties of immature particles. This suggests that individual patients develop different responses to prM. On the basis of these results, we believe that it is important to further investigate the antibody responses in DENV-infected patients and to unravel if patients with prM antibodies are more susceptible to develop severe disease. In this respect, it is interesting to note that the rates of prM antibody responses are significantly higher in patients experiencing a secondary infection compared to a primary infection [23]. Clearly, future clinical studies are required to obtain further evidence for the role of immature particles and prM antibodies in disease development.

## Materials and Methods

### Cells

*Aedes albopictus* C6/36 cells were maintained in minimal essential medium (Life Technologies) supplemented with 10% fetal bovine serum (FBS), 25 mM HEPES, 7.5% sodium bicarbonate, penicillin (100 U/ml), streptomycin (100 µg/ml), 200 mM glutamine and 100 µM nonessential amino acids at 28°C, 5% CO_2_. Baby Hamster Kidney-21 clone 15 cells (BHK-15) cells were cultured in DMEM (Life Technologies) containing 10% FBS, penicillin (100 U/ml), streptomycin (100 µg/ml), 10 mM HEPES, and 200 mM glutamine. Human adenocarcinoma LoVo cells were cultured in Ham's medium (Life Technologies) supplemented with 20% FBS at 37°C, 5% CO_2_. Human erythroleukemic K562 cells were maintained in DMEM supplemented with 10% FBS, penicillin (100 U/ml), and streptomycin (100 µg/ml) at 37°C, 5% CO_2_. Human leukemic monocyte lymphoma U937 cells were maintained in Iscove's modified Dulbecco's medium (GIBCO) supplemented with 10% FBS, 4 mM L-glutamine, penicillin (100 U/ml), and streptomycin (100 µg/ml) and adjusted to contain 1.5 g/l sodium bicarbonate, 10 mM HEPES and 1.0 mM sodium pyruvate (GIBCO). Cells were incubated at 37°C at 5% CO_2._ Human peripheral blood mononuclear cells (PBMCs) were maintained in RPMI 1640 medium supplemented with 10% FBS, penicillin (100 U/ml), and streptomycin (100 µg/ml). PBMCs were isolated from heparinized blood samples collected from healthy persons using standard density centrifugation procedures with Lymphoprep™ (AXIS-SHIELD). The PBMCs were used immediately after isolation or cryopreserved at −150°C. On the day of infection, the percentage of CD14+, CD19- population within isolated PBMCs was determined (5%–10% depending on the blood donor) using cell surface markers CD-14 -FITC and CD19-R-PE purchased from commercial source (IQ Products).

### Virus growth

DENV-2 strain 16681, kindly provided by dr. Claire Huang (Center for Disease Control and Prevention, USA), was propagated on C6/36 cells as described before [16]. Briefly, monolayer of C6/36 cells was infected at multiplicity of infection (MOI of 0.1). At 96 hpi, the medium was harvested, cleared from cellular debris by low-speed centrifugation, aliquoted, and stored at −80°C. Immature DENV particles were produced on LoVo cells as described previously [16]. Briefly, LoVo cells were infected at MOI 10. Virus inoculum was removed after 1.5 h and fresh medium was added after washing the cells twice with PBS. At 72 hpi, the medium containing the virus particles was harvested, cleared from cellular debris by low-speed centrifugation, aliquoted, and stored at −80°C. [^35^S] methionine-labeled immature virus was prepared, as described previously [16]. Briefly, cells were infected at a MOI of 10. At 2 hpi, 400 µCi of [^35^S]methionine (Amersham Biosciences) was added to 20 ml of medium and incubation was continued overnight. At 23 hpi, the medium was supplemented with an additional 200 µCi of radioactive label. At 72 hpi, the supernatant containing the viral particles was cleared from cell debris by low-speed centrifugation and the virions were pelletted and further purified on a discontinuous (20 and 55% w/v) Optiprep™ gradient (Axis-Shield) by ultracentrifugation. Virus was harvested from the gradient interface, aliquoted and stored at −80°C. Virus preparations were analyzed with respect to the infectious titer and the number of genome-containing particles, as described previously [15],[16].

The furin-cleavage mutant (pDENprMΔ90) was generated by deletion of the lysine codon within the furin-recognition site at position 90 of prM. The mutation was introduced in the DENV-2 16681 infectious cDNA clone (pD2/IC-30P) [35]. Briefly, two PCR fragments were generated using the following primers: forward primer A (5′-CTC AAC GAC AGG AGC ACG ATC AT- 3′) and reverse primer A (5′- GAG TGC CAC TGA TCT TTC TCT TC-3′) and forward primer B (5′- GAA GAG AAA Δ GAT CAG TGG CAC TCG TT-3′) and reverse B (5′-GTG TCA TTT CCG ACT GCA TGC TCT-3′). The PCR fragments were ligated, cut with SacI and Sph1, and ligated into pD2/IC-30P. The introduced deletion was confirmed by DNA sequence analysis using an automated capillary sequencing system (ABI). RNA transcripts were generated from pDENprMΔ90 and pD2/IC-30P using T7 RNA polymerase and transfected into BHK-15 cells cells by electroporation (Bio-Rad Gene Pulser apparatus; two pulses at 1.5 kV, 25 µF, and 200 Ω). At 12 hours post transfection (hpt) cells were washed extensively to remove remaining RNA copies. Virus preparations were harvested at 72 hpt, cleared from cellular debris by low-speed centrifugation, aliquoted, and stored at −80°C. Virus preparations were analyzed with respect to the infectious titer and the number of genome-containing particles, as described previously. The antigenic reactivity of DENVprMΔ90 was compared to LoVo-derived virus by standard three-layer ELISA. Briefly, microtiter ELISA plates (Greiner bio-one) were coated with 4×10^6^ GCP of different virus preparations per well in 100 µl coating buffer, overnight. After blocking with 2% milk in coating buffer for 45 min, 100 µl of two-fold serial dilutions of anti-DENV mAbs were applied to the wells and incubated for 1.5 h, in triplicate. Subsequently, 100 µl of horseradish peroxidase-conjugated goat anti-mouse IgG-isotype antibody (Southern Biotech) was applied for 1 h. All incubations were performed at 37°C. Staining was performed using o-phenylene-diamine (OPD) (Eastman Kodak Company) and absorbance was read at 492 nm (A_492_) with an ELISA reader (Bio-tek Instruments, Inc.).

### Patient sera

Convalescent sera from DENV-2 immune, hospitalized patients were kindly provided by dr. G. Comach (Biomed-UC, Lardidev, Maracay, Venezuela) and dr. T. Kochel (U.S. Naval Medical Research Center Detachment, Lima, Peru). All sera samples analyzed were obtained between 20–28 days following DENV-infection.

### Infectivity assays

Virus or virus-antibody complexes were added to 2×10^5^ K562 cells, at a multiplicity of 100 genome-containing particles (MOG) per cell. After 1.5 h incubation at 37°C, the inoculum was removed and fresh medium was added to the cells. At 24–48 hpi, the medium was harvested and virus production was analyzed by plaque assay on BHK-15 cells, as described previously [36]. To measure the number of infected cells, cells were fixed at 24–48 hpi, stained with 3H5-conjugated Alexa647, and analyzed using a FACS Calibur cytometer. For virus-antibody complex formation, virus particles were incubated for 1 h at 37°C with various dilutions of monoclonal prM antibody 70-21 and 2H2 in cell culture medium containing 2% FBS prior to the addition to cells. To investigate the involvement of the Fc receptor, mAb 70–21 F(ab')_2_ fragments produced by use of the immobilized pepsin (Pierce) were used. Alternatively, K562 cells were pretreated with 25 µg/ml of anti-FcγRII antibody (MCA1075PE, Serotec) for 1 h at 37°C, after which access antibody was removed by extensive washing. In furin blockage experiments, cells were treated with 25 µM of furin-specific inhibitor, decanoyl-L-arginyl-L-valyl-L-lysyl-L-arginyl-chloromethylketone (decRRVKR-CMK) (Calbiochem) prior and during virus infection. In control sample for the decRRVKR-CMK activity, additional 25 µM of the inhibitor was added to ensure blockage of the progeny virus maturation.

### Binding assays

To determine the number of bound genome-containing particles per cell, virus or virus-antibody complexes were incubated with 2×10^5^ K526 cells at MOG 1000 for 1 h at 4°C. Subsequently, cells were washed three times with ice-cold PBS containing MgCl_2_ and CaCl_2_ (Life Technologies) to remove unbound virus-antibody complexes. Then, viral RNA was extracted from the cells by use of the QIAamp Viral RNA mini Kit (QIAGEN). Thereafter, cDNA was synthesized from the viral RNA with reverse transcription-PCR (RT-PCR), copies of which were quantified using quantitative PCR [15].

### In vitro furin cleavage assays

[^35^S]methionine-labeled immature particles or viral immune complexes were incubated with furin [New England BioLabs] for 16 h at pH 6.0, as described previously [16]. Following furin treatment samples were subjected to sodium dodecyl sulphate-polycrylamide gel electrophoresis (SDS-PAGE) analysis to visualize the protein composition.

## Supporting Information

Figure S1 Time course of the number of DENV-infected K562 cells. Cells were infected with wild-type DENV at MOG 100. At the indicated time points, cells were fixed, stained intracellularly with Alexa-647-coupled anti-E antibody 3H5.1, and subjected to flow-cytometric analysis. MFI denotes “mean fluorescence intensity” of infected cells.
(0.01 MB PDF)

Figure S2 prM antibody 2H2 enhances the infectious properties of immature DENV particles. Cells were infected with immature (prM) or wild-type (wt) DENV-2 particles at MOG 100 in the presence or absence of anti-prM 2H2. Virus particle production was measured at 43 hpi by plaque assay on BHK-15 cells. (A) K562 cells, (B) U937 cells, (C) PBMCs. Data are expressed as means of at least two independent experiments. The error bars represent standard deviations (SD); (n.d.) denotes “not detectable”.
(0.01 MB PDF)

Figure S3 prM antibody 2H2 enhances the infectious properties of wild-type DENV in various cell types. Cells were infected with wild-type (wt) DENV-2 at MOG 100 in the presence of increasing concentrations of 2H2. Virus particle production was measured at 43 hpi by plaque assay on BHK-15 cells. (A) K562 cells, (B) U937 cells, (C) PBMCs. Data are expressed as means of at least three independent experiments. The error bars represent standard deviations (SD); (n.d.) denotes “not detectable”; * denotes significance (p<0.05) analyzed using Two-tailed Student's t-tests.
(0.01 MB PDF)

Figure S4 DENV-immune sera stimulate infectivity of wild-type DENV. U937 cells were infected with wild-type (wt) DENV at MOG 100 in the presence of 10-fold sequential dilutions of polyclonal sera. Virus particle production was measured at 43 hpi by plaque assay on BHK-15 cells. Viral titers obtained at 10^4^ sera dilution are depicted on the plot. The error bars represent standard deviations (SD).
(0.01 MB PDF)
