# Supplementary figures and images for: Immature Dengue Virus: A Veiled Pathogen?
Source: PLoS Pathog. 2010 Jan 8;6(1):e1000718. doi: 10.1371/journal.ppat.1000718 (PMC2798752; doi:10.1371/journal.ppat.1000718)

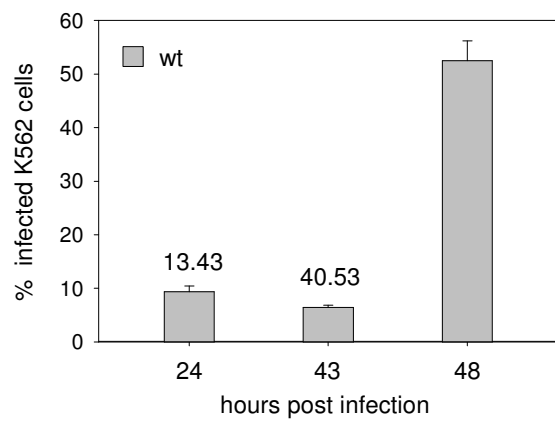

**Fig. S1**

Supplement: Figure S1 — Time course of the number of DENV-infected K562 cells. Cells were infected with wild-type DENV at MOG 100. At the indicated time points, cells were fixed, stained intracellularly with Alexa-647-coupled anti-E antibody 3H5.1, and subjected to flow-cytometric analysis. MFI denotes “mean fluorescence intensity” of infected cells. (0.01 MB PDF) [file ppat.1000718.s001.pdf]

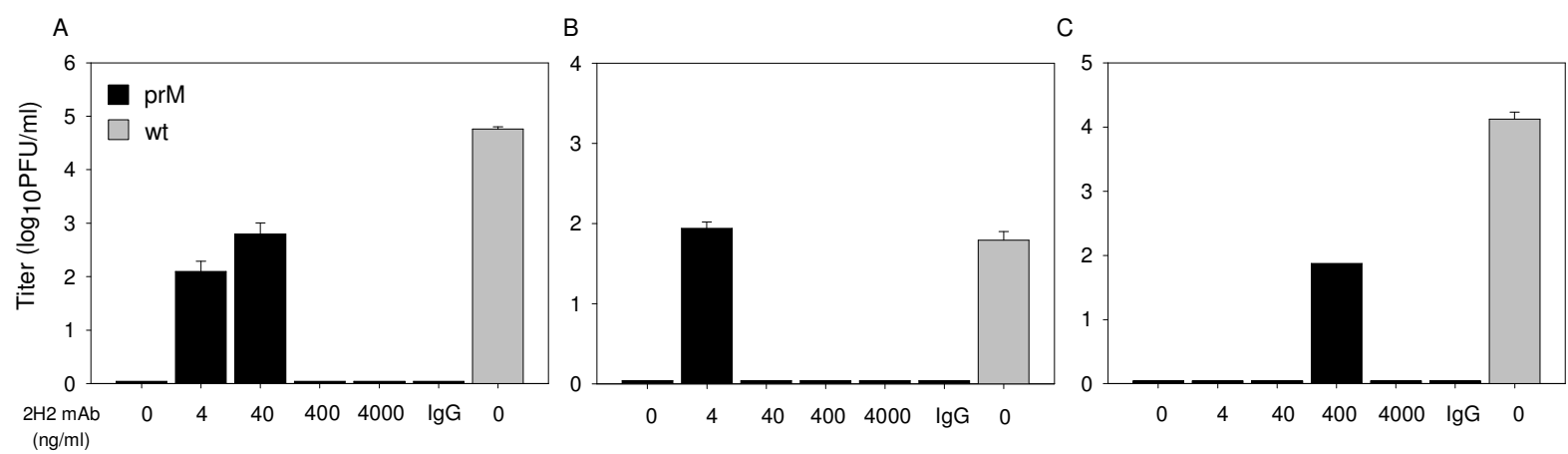

**Fig. S2**

Supplement: Figure S2 — prM antibody 2H2 enhances the infectious properties of immature DENV particles. Cells were infected with immature (prM) or wild-type (wt) DENV-2 particles at MOG 100 in the presence or absence of anti-prM 2H2. Virus particle production was measured at 43 hpi by plaque assay on BHK-15 cells. (A) K562 cells, (B) U937 cells, (C) PBMCs. Data are expressed as means of at least two independent experiments. The error bars represent standard deviations (SD); (n.d.) denotes “not detectable”. (0.01 MB PDF) [file ppat.1000718.s002.pdf]

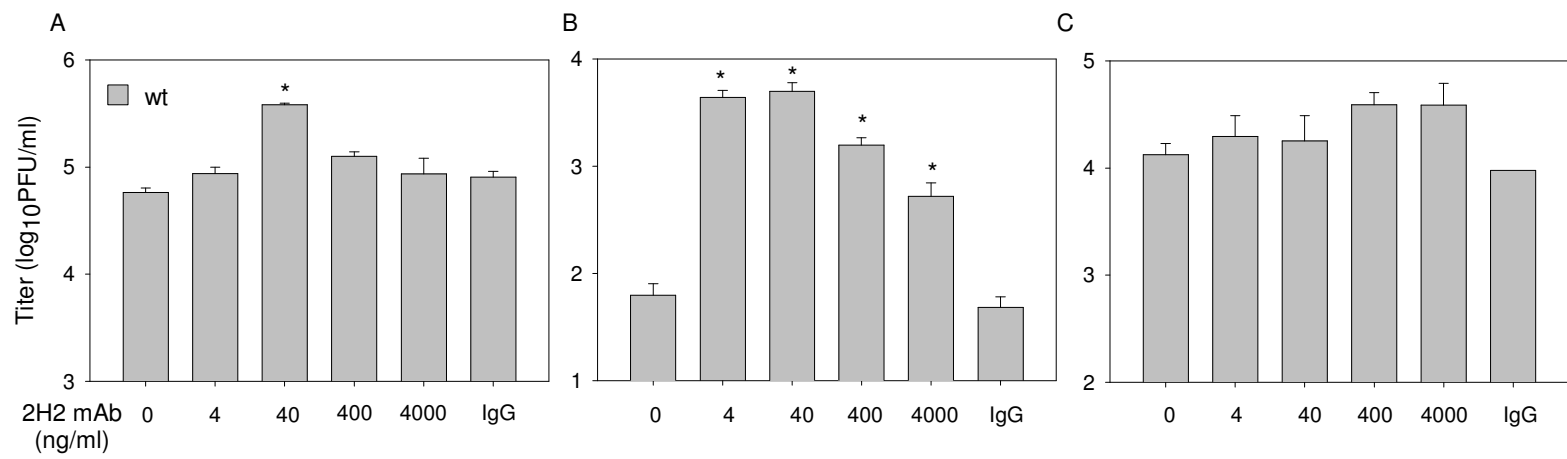

**Fig. S3**

Supplement: Figure S3 — prM antibody 2H2 enhances the infectious properties of wild-type DENV in various cell types. Cells were infected with wild-type (wt) DENV-2 at MOG 100 in the presence of increasing concentrations of 2H2. Virus particle production was measured at 43 hpi by plaque assay on BHK-15 cells. (A) K562 cells, (B) U937 cells, (C) PBMCs. Data are expressed as means of at least three independent experiments. The error bars represent standard deviations (SD); (n.d.) denotes “not detectable”; * denotes significance (p<0.05) analyzed using Two-tailed Student's t-tests. (0.01 MB PDF) [file ppat.1000718.s003.pdf]

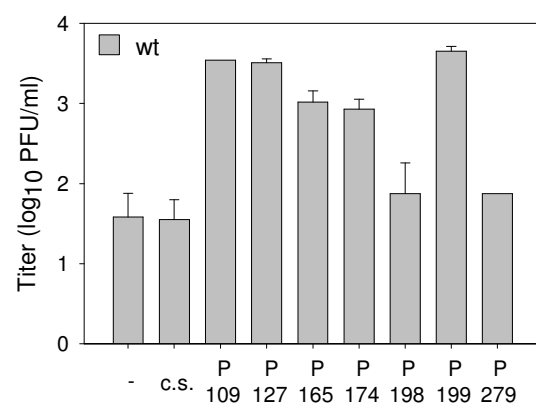

**Fig. S4**

Supplement: Figure S4 — DENV-immune sera stimulate infectivity of wild-type DENV. U937 cells were infected with wild-type (wt) DENV at MOG 100 in the presence of 10-fold sequential dilutions of polyclonal sera. Virus particle production was measured at 43 hpi by plaque assay on BHK-15 cells. Viral titers obtained at 104 sera dilution are depicted on the plot. The error bars represent standard deviations (SD). (0.01 MB PDF) [file ppat.1000718.s004.pdf]
